# Integrative analysis of multi-omics data reveals distinct impacts of DDB1-CUL4 associated factors in human lung adenocarcinomas

**DOI:** 10.1038/s41598-017-00512-1

**Published:** 2017-03-23

**Authors:** Hong Yan, Lei Bi, Yunshan Wang, Xia Zhang, Zhibo Hou, Qian Wang, Antoine M. Snijders, Jian-Hua Mao

**Affiliations:** 1 Department of Laboratory Medicine, Nanjing Chest Hospital, 215 Guangzhou Road, Nanjing, 210029 China; 2 Biological Systems and Engineering Division, Lawrence Berkeley National Laboratory, Berkeley, California, USA; 3 School of Preclinical Medicine, Nanjing University of Chinese Medicine, 138 Xianlin Road, Nanjing, 210023 China; 4 International Biotechnology R&D Center, Shandong University School of Ocean, Weihai, Shandong, 264209 China; 5 Department of Tuberculosis, Nanjing Chest Hospital, 215 Guangzhou Road, Nanjing, 210029 China; 6 Department of First Respiratory, Nanjing Chest Hospital, 215 Guangzhou Road, Nanjing, 210029 China

## Abstract

Many DDB1-CUL4 associated factors (DCAFs) have been identified and serve as substrate receptors. Although the oncogenic role of CUL4A has been well established, specific DCAFs involved in cancer development remain largely unknown. Here we infer the potential impact of 19 well-defined DCAFs in human lung adenocarcinomas (LuADCs) using integrative omics analyses, and discover that mRNA levels of DTL, DCAF4, 12 and 13 are consistently elevated whereas VBRBP is reduced in LuADCs compared to normal lung tissues. The transcriptional levels of DCAFs are significantly correlated with their gene copy number variations. SKIP2, DTL, DCAF6, 7, 8, 13 and 17 are frequently gained whereas VPRBP, PHIP, DCAF10, 12 and 15 are frequently lost. We find that only transcriptional level of DTL is robustly, significantly and negatively correlated with overall survival across independent datasets. Moreover, DTL-correlated genes are enriched in cell cycle and DNA repair pathways. We also identified that the levels of 25 proteins were significantly associated with DTL overexpression in LuADCs, which include significant decreases in protein level of the tumor supressor genes such as PDCD4, NKX2-1 and PRKAA1. Our results suggest that different CUL4-DCAF axis plays the distinct roles in LuADC development with possible relevance for therapeutic target development.

## Introduction

Lung cancer is the leading cause of death from cancer in the United States resulting in over 150,000 deaths per year^1, 2^, where non-small cell lung cancer is the predominant form of the disease. Despite major advances in our knowledge of the genetic factors involved in lung cancer through detailed molecular analysis using DNA and RNA microarrays and next generation sequencing, the 5 year survival rate for Non-Small Cell Lung cancer (NSCLC) patients is approximately 21%^3^. Lung cancer treatment is therefore moving rapidly towards an era of personalized medicine, where the molecular characteristics of a patient’s tumor will dictate the optimal treatment modalities. NSCLC patients with EGFR mutations show significantly improved responses to treatment with Tyrosine kinase inhibitors, e.g., gefitinib or erlotinib that target this receptor kinase^4^. However, almost all of these patients eventually relapse due to development of resistance through various mechanisms^5^. This paradigm of tumor rewiring in the face of targeted treatment is evident from many clinical trials that have been reported, suggesting that the identification of complementary targets will be necessary to improve survival and the probability of long term cures.

The ubiqutin protease system (UPS) regulates a variety of basic cellular pathways associated with cancer development^6^. Not surprisingly, aberrations in the UPS are implicated in the pathogenesis of various human malignancies^7, 8^. The cullin-RING ligases (CRLs) are the largest E3 ligase family involved in the UPS. In mammals, there are seven different cullins (CUL1, 2, 3, 4A, 4B, 5 and 7) and two rings (RBX1 and 2)^9^. The activity of cullin-dependent E3 ligases is regulated through the reversible attachment of an ubiqutin-like Nedd8 protein. Post-translational modifications of these cullins by Nedd8 are essential for their association with the E2 enzyme, and then they can recruit various substrate proteins for their ubiquitin-mediated degradation^10^.

*CUL4A* amplification or overexpression has been reported in many types of human cancer, including breast cancer, squamous cell carcinoma, adrenocortical carcinoma, childhood medulloblastoma, prostate cancer and hepatocellular carcinoma and overexpression contributes to tumor progression, metastasis, and poor prognosis of cancer patients^11–18^. A recent study suggests that CUL4A is a promising therapeutic target and a potential biomarker for EGFR targeted therapy in NSCLC patients^19^. The use of a *Cul4A* transgenic mouse model further demonstrated the potential oncogenic role of *Cul4A* in lung tumor development. After 40 weeks of Cul4A overexpression, lung tumors were visible and were characterized as grade I or II adenocarcinomas^20^. In contrast, *Cul4A* knockout mice are resistant to UV induced skin carcinogenesis^15^. All evidence indicates that *CUL4A* is a critical oncogene in cancer development.

Recently, many DDB1-CUL4 associated factors (DCAFs) have been identified and serve as substrate receptors to execute the degradation of proteins^21, 22^. However, the specific CUL4A-DCAF nexus that contributes to human cancer development remains largely unknown. In this study, we combined available cancer databases to infer the potential impact of 19 well-defined DCAFs in human lung adenocarcinomas (LuADCs) by combining gene transcript and DNA copy number variations with their ability to predict patient prognosis. Our strategy provides further evidence for a critical role of DCAFs in LuADC development, which could be exploited to design better treatment strategies.

## Results

### DECAFs are differentially expressed in LuADCs

**Figure 1:**
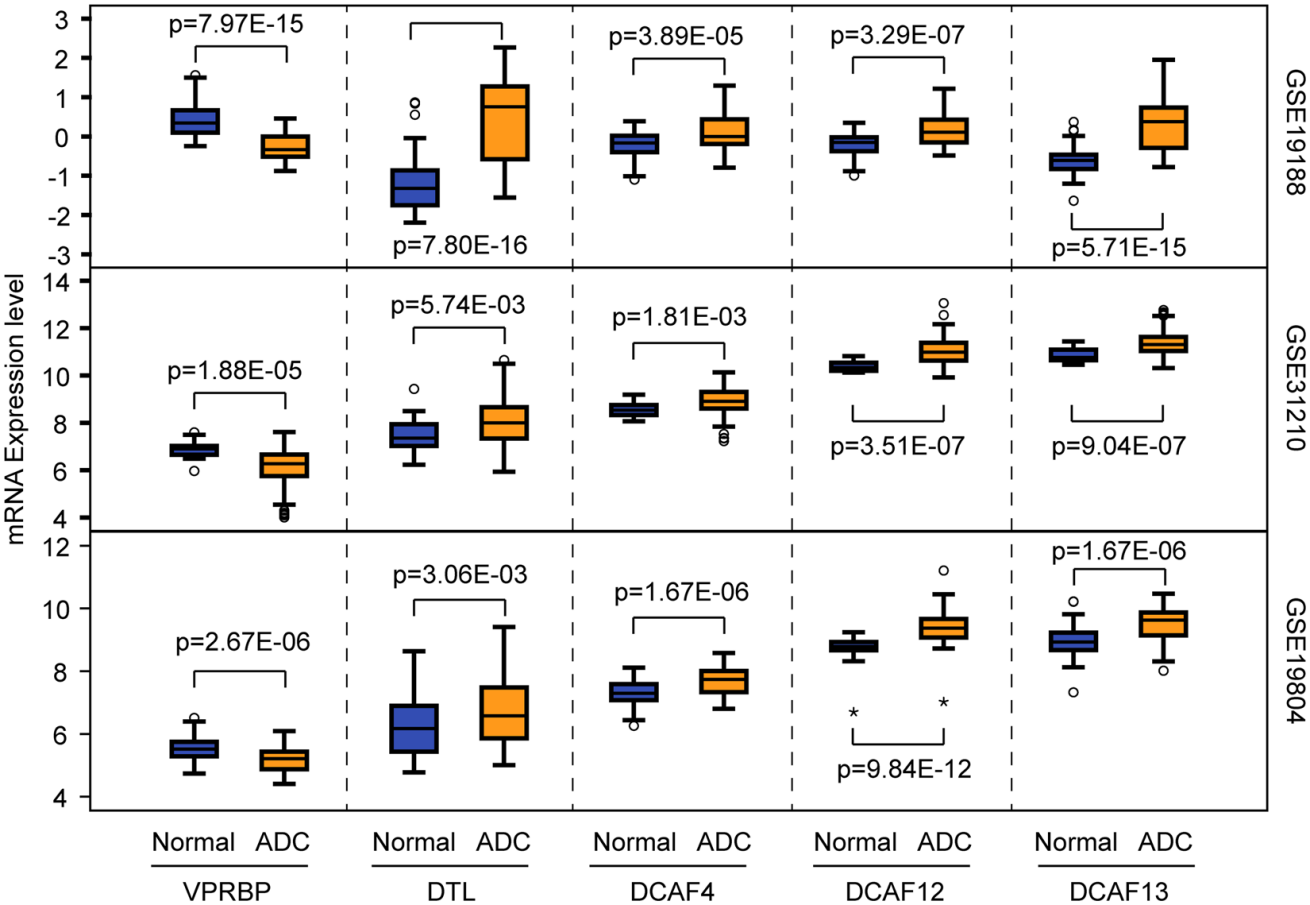
*VPRBP*, *DTL*, *DCAF4*, *DCAF12* and *DCAF13* are consistently deregulated in lung adenocarcinoma. *VPRBP* gene expression was significantly downregulated, whereas *DTL*, *DCAF4*, *DCAF12* and *DCAF13* were significantly upregulated in LuADCs (indicated in orange; GSE19188: N = 45; GSE31210: N = 226; GSE19804: N = 60) compared to expression in normal lung tissue (indicated in blue; GSE19188: N = 65; GSE31210: N = 20; GSE19804: N = 60). Increased or decreased gene expression cut-off: 2-fold and adjusted p < 0.05.

To gain insight into the role of 19 well-defined DCAFs (Table S1) in human LuADCs, we investigated whether the expression of DCAFs is altered in LuADCs compared to normal lung tissues using 3 independent datasets (GSE19188, GSE31210 and GSE19804). The significant differential expression of all DCAFs was assessed by a cut-off of two-fold change and adjusted p-value < 0.05 (Table S1). We found that the transcriptional epxression level of *VPRBP* was consistently reduced whereas transcriptional expression levels of *DTL*, *DCAF4*, *12* and *13* were consistently elevated in LuADCs (Fig. 1, Table S1). The changes in the expression of the remaining DCAFs were found to be insignificant and/or inconsistent across the three datasets (Table S1).

### Transcriptional levels of DCAFs are correlated with their copy number variations

**Figure 2:**
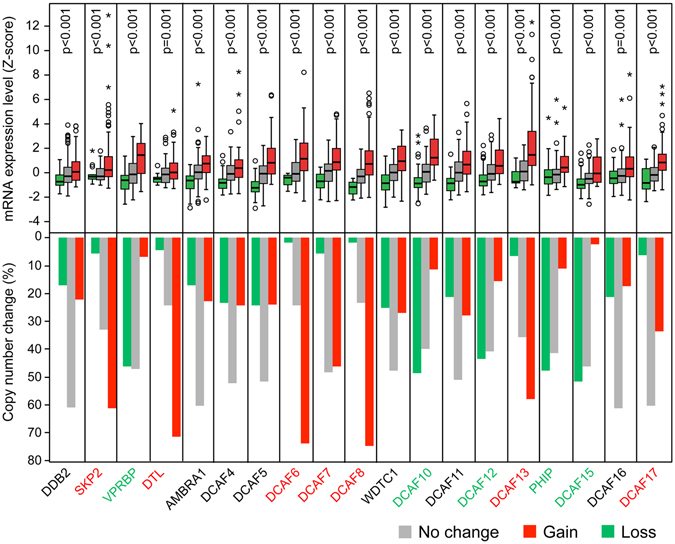
DCAF gene expression is strongly correlated with its DNA copy number in lung adenocarcinoma. The relationship between tumor DNA copy number and gene expression for 19 DCAFs in LuADCs is shown in top panel. Significance was determined using Kruskal-Wallis test. Bottom panel shows the percent of tumors with DNA copy number change. Tumors with increased DNA copy number (Gain) are indicated in red and those with decreased DNA copy number (Loss) in green. Tumors with no change in DNA copy number are indicated in gray.

The Cancer Genome Atlas (TCGA) data were analyzed to search for the possible mechanism by which the expression of DCAFs is altered in LuADCs. Whereas mutations in DCAFs were relatively rare (Figure S1), DNA copy number alterations encompasing DCAFs were frequently observed in LuADCs (Fig. 2, bottom panel). *SKIP2*, *DTL*, *DCAF6*, *7*, *8*, *13* and *17* are frequently gained whereas *VPRBP*, *PHIP*, *DCAF10*, *12* and *15* are frequently lost (Fig. 2, bottom panel). Changes in DNA copy number are often observed in tumors, and are one mechanism that can result in a change in gene expression during tumor progression. Therefore, we investigated whether the transcriptional expression levels of DCAFs are significantly correlated with their copy number variations using a rank-based nonparametric test. Indeed, there was a significant correlation between DNA copy number and transcriptional expression for all DCAFs (Fig. 2, upper panel).

### Increased expression of *DTL* is consistently associated with poor survival

**Figure 3:**
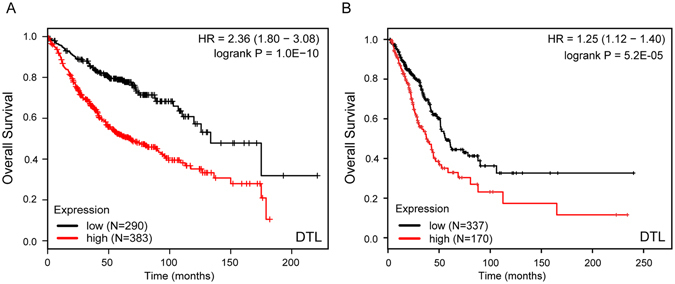
*DTL* gene expression is associated with overall survival in LuADCs. Survival risk curves are shown for *DTL* expression in LuADCs using KM-plotter (probe ID 222680_s_at) (**A**) and TCGA (**B**). Low and high expression level of *DTL* are drawn in black and red respectively. In KM-plotter the threshold for the high and low DTL expression cohorts is automatically calculated. In TCGA, patients were divided into tertiles based on DTL expression levels. The top tertile was defined as the high DTL expression cohort and the remaining patients were defined as the low DTL expression cohort. The p-value represents the equality of survival curves based on a log-rank test.

To further assess the importance of DCAFs in LuADC development, we next evaluated their prognostic value for LuADC patients in a large public clinical microarray database using the Kaplan-Meier plotter (http://kmplot.com/analysis/index.php?p=service&cancer=lung)^23^. Patients were divided into two groups based on the expression levels of each individual DCAF. Subsequently, the effect of high or low expression level of these DCAFs on the overall survival (OS) was evaluated using Cox regression analysis, the Kaplan-Meier survival curve and log-rank test. From the log-rank test p-values and hazard ratio (HR) derived from univariate analysis (Table S2), we found that high transcriptional levels of *DTL* and *DCAF15* are significantly associated with shortened overall survival (OS) whereas high transcriptional levels of *DDB2*, *DCAF4* and *DCAF12* favor good prognosis (Table S2). Using TCGA data, we reevaluated the impact of DCAFs on OS and only confirmed that high transcriptional level of DTL is significantly associated with poor OS (Fig. 3, Table S2), suggesting that DTL is a robust biomarker for prognosis among DCAFs.

### Bioinformatics analysis infers potential mechanisms of DTL functions in LuADC development

**Figure 4:**
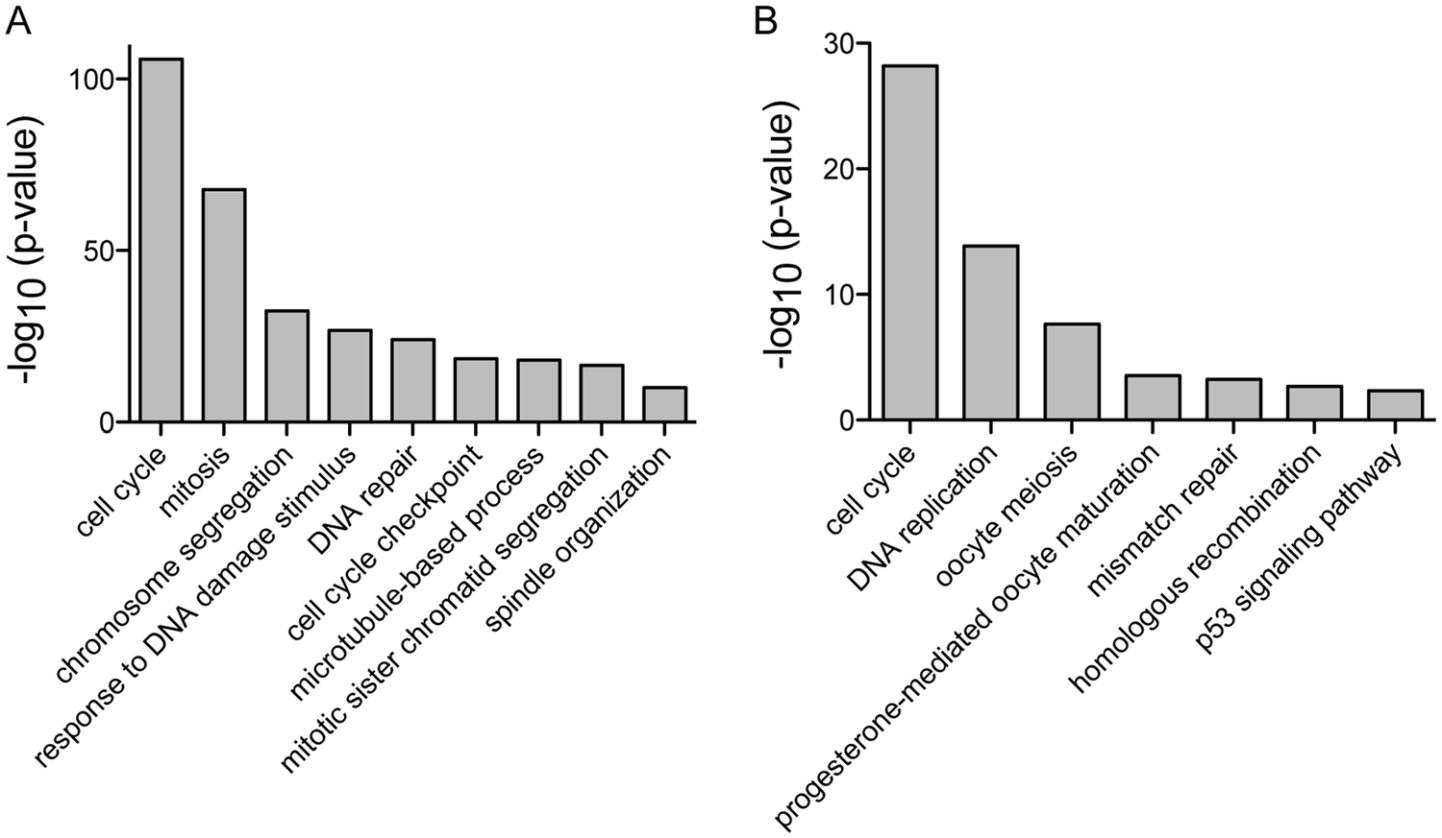
Genes transcriptionally co-expressed with DTL in LuADCs are significantly enriched for cell cycle and DNA repair pathways by Gene Ontology ﻿(**A**) and KEGG pathway (**B**) analysis.

**Figure 5:**
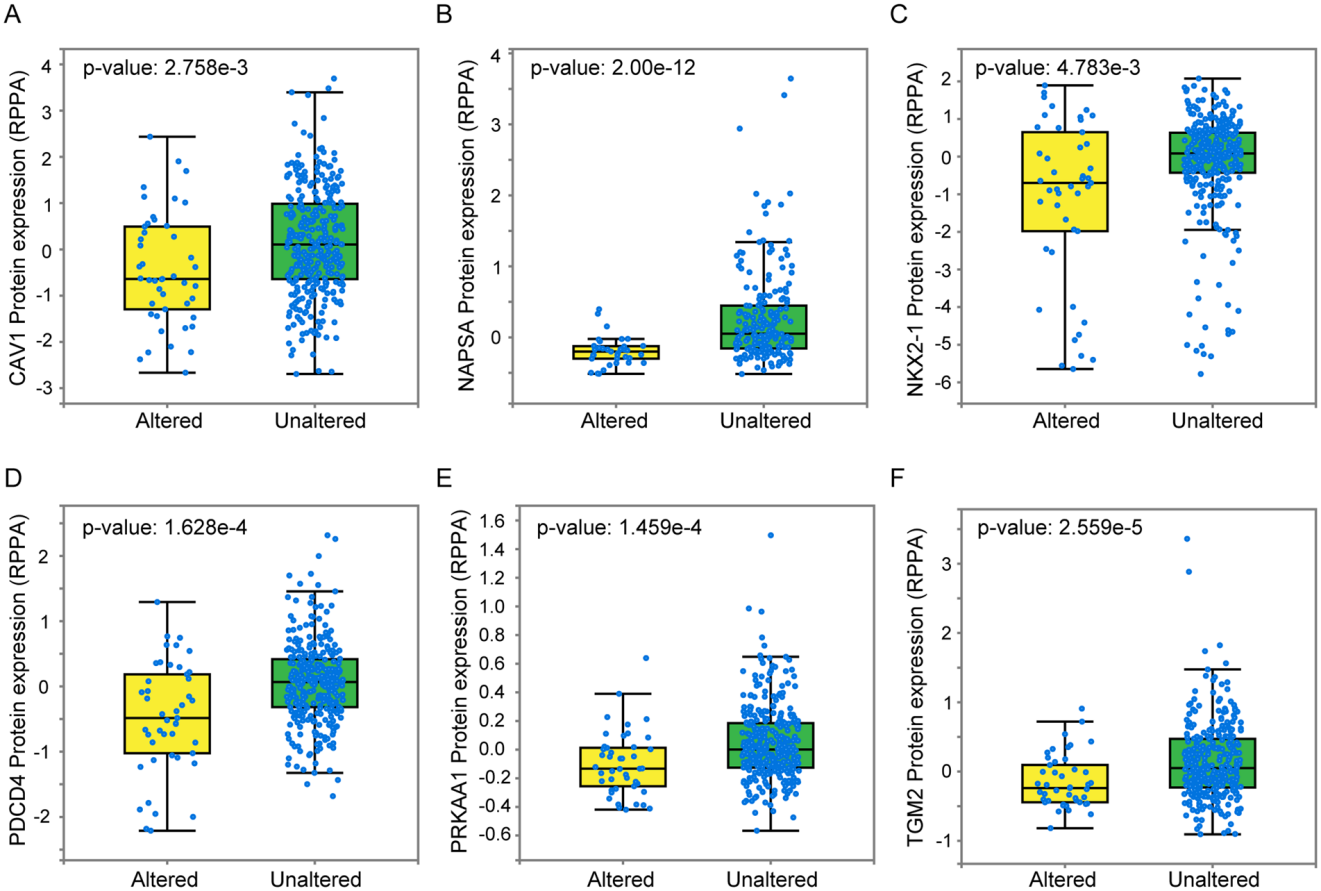
Significantly altered protein expression profiles in DTL overexpressing lung adenocarcinoma. Protein expression of CAV1 (**A**), NAPSA (**B**), NKX2-1 (**C**), PDCD4 (**D**), PRKAA1 (**E**) and TGM2 (**F**) in LuADCs overexpressing DTL (Altered) compared to tumors that do not overexpress DTL (Unaltered).

To search for the mechanisms by which DTL overexpression facilitated tumor development, we first identified the genes that are transcriptionally co-expresssed with DTL in LuADC using TCGA data. Correlation analysis revealed that 257 genes were signficantly co-expressed with DTL (Spearman rank R > 0.6 or <−0.6; Table S3). 254 of them are positivly correlated with DTL. Gene ontology and KEGG pathway analysis revealed that genes correlated in expression with DTL are enriched in cell cycle and DNA repair pathways (Fig. 4, Table S4). Interestingly, all these cell cycle and DNA repair genes were co-overexpressed with DTL (Table S3). Finally, we investigated whether protein expression of any gene was significantly different between the LuADC group that overexpress DTL versus those without DTL overexpression. A significant difference was observed in the expression of 25 proteins (Table S5; adjusted p < 0.05). For example, protein levels of CCNB1 (cyclin B1), CCNE1 and 2, and PCNA were significantly higher in LuADCs overexpressing DTL (Table S5). In contrast, protein levels of CAV1 (p = 2.76E-03), NAPSA (p = 2.00E-12), NKX2-1 (p = 4.78E-03), PDCD4 (p = 1.63E-04), PRKAA1 (p = 1.46E-04), and TGM2 (p = 2.56E-05) were signiciantly lower in LuADCs overexpressing DTL (Fig. 5). PDCD4, NKX2-1 and PRKAA1 (AMPKA1) are well known tumor supressor genes, and our result suggests that DTL downregulates these genes.

## Discussion

Accumulating evidence has demonstrated that CUL4A plays a critical role in cancer development^11–18^. CUL4A composes the multifunctional ubiquitin ligase E3 complex where specific DCAFs determine substrate specificity. A number of DCAFs have been identified recently^21, 22^. In this study, we used a systematic multi-omics approach to assess oncogenic properties of DCAFs in human LuADCs. We found that in comparison to normal lung tissues, the transcriptional levels of VPRBP are robustly downregulated in LuADCs across three datasets, which is consistent with frequent loss of VPRBP DNA copy number. In contrast, DTL and DCAF13 were frequently gained and their transcriptional levels robustly elevated. These results suggest that in LuADCs VPRBP (DCAF1) may act as a tumor suppressor gene whereas DTL and DCAF13 act as oncogenes.

Not much is known about the role of DCAF13 in tumorigenesis. In contrast, DTL has previously been shown to play an oncogenic role in different cancer types including gastric cancer, ewing sarcoma, melanoma and ovarian cancer^24–27^. DTL is located on chromosome 1q, which is often gained in different tumor types. In Ewing sarcoma, 1q gain was associated with poor patient survival and DTL was significantly overexpressed in tumors with 1q gain^27^. In gastric cancer, DTL was also overexpressed compared to normal gastric tissue^24^. Knockdown of DTL in gastric cancer cells induced apoptosis and G2/M arrest whereas overexpression of DTL promoted anchorage-independent growth. More recently, it was suggested that DTL is not an oncogene, but rather a gene which tumor cells become addicted to in order to control enhanced tumor cellular stress such as replicative stress and DNA damage^28^. Consistent with this, studies in zebrafish showed that DTL promotes genomic stability by controlling CDT1 levels preventing rereplication and by controlling the early G2/M checkpoint^29^.

Our data indicated that VPRBP DNA copy number was frequently lost and transcript levels were down-regulated in LuADCs. This suggests that VPRBP (DCAF1) may act as a tumor suppressor gene in contrast to prior reports, which showed an oncogenic role for DCAF1^30^. Survival analysis for VPRBP in LuADCs was inconsistent, low expression of VPRBP probe ID 204377_s_at was significantly associated with poor patient survival (p = 7.9E-07), whereas probe ID 204376_at showed only a borderline association with patient survival (p = 0.055). To further investigate if this was also observed in other tumor types, we tested the association of VPRBP transcript and overall survival in breast, ovarian and gastric cancer. Similar to LuADCs, low VPRBP transcript levels were associated with poor survival in breast cancer patients (p < 1.6E-06). In contrast, low VPRBP transcript levels were associated with good prognosis in gastric cancer patients (p < 0.03). No association with patient survival was observed in ovarian cancer. Taken together, these data suggest that the role of VPRBP appears to be context dependent. Could it be possible that tumor cells select for low/moderate levels of VPRBP to prevent uncontrollable overproliferation and subsequent exhaustion of survival factors? Further research will be needed to clarify the role of VPRBP in tumorigenesis.

In conclusion, our multi-omics analysis of DCAFs reveals distinct impacts of DCAFs in human LuADCs, which opens up a new horizon for further understanding the significance of DCAF genes in LuADC development and clinical applications in diagnosis, prognosis and therapy for LuADC patients.

## Materials and Methods

### Datasets used in study

A comprehensive search for DCAF genes was conducted using the UniGene and GeneCard databases and literature, which resulted in 19 DCAFs that were used in our study (Table S1). Gene transcript data of normal and tumor tissues was obtained from The National Center for Biotechnology Information (NCBI) The Gene Expression Omnibus (GEO) (GSE31210, GSE19804 and GSE19188). The processed data for each of these three data sets were obtained from the GEO website for analysis. The criteria for inclusion in our analysis were that all samples had to be analyzed using Affymetrix U133A and B or U133 plus2.0, which included all genes in the human genome. We furthermore required that each study included both tumor tissue and normal tissue analyzed simultaneously. All tumor samples for our analysis are lung adenocarcinomas (ADC). Genomic DNA copy number alterations and mRNA expression levels of DCAFs in LuADC were obtained from cBioPortal (TCGA)^31, 32^. All information concerning these samples can be downloaded from cBioPortal (http://www.cbioportal.org/data_sets.jsp).

### Statistical analysis

GEO2R was used to identify the differentially expressed DCAFs between normal and tumor tissues in GSE31210, GSE19804 and GSE19188 (adjusted p-value ≤ 0.05 and fold changes ≥ 2). We used a rank-based nonparametric test (Kruskal-Wallis) to determine whether gene expression levels were significantly different between three copy number groups (gain: increased copy number, loss: decreased copy number and no change: no change in copy number; p < 0.01 was used as a threshold for significance).

The association of DCAF genes with overall survival of LuADC patients was assessed using the Kaplan-Meier plotter using all available patients restricted to adenocarcinoma histological subtype (http://kmplot.com/analysis/index.php?p=service&cancer=lung)^23^. The best performing threshold between the upper and lower quartiles was used as a threshold for separating the patient cohort into low and high expression groups (Table S3). For TCGA data, we first used univariate Cox regression to investigate which DCAFs were significantly associated with overall survival (Table S2). Kaplan-Meier plots were constructed and a long-rank test was used to determine differences among overall survival according to *DCAFs* mRNA levels. All analyses were performed by SPSS 11.5.0 for Windows. A two-tailed p-value of less than 0.05 was considered to indicate statistical significance.

Gene ontology enrichment analysis was performed using the web based gene set analysis toolkit (BH corrected p < 0.05 was used as a threshold for significance)^33, 34^.

## Electronic supplementary material

Supplementary Figure and tables
