# Supplementary material for: Integrative analysis of multi-omics data reveals distinct impacts of DDB1-CUL4 associated factors in human lung adenocarcinomas
Source: Sci Rep. 2017 Mar 23;7:333. doi: 10.1038/s41598-017-00512-1 (PMC5428704; doi:10.1038/s41598-017-00512-1)
Supplement: Supplementary file 1 — Supplementary Figure and tables [file 41598_2017_512_MOESM1_ESM.pdf]

Integrative analysis of multi-omics data reveals distinct impacts of DDB1-CUL4 associated factors in human lung adenocarcinomas

Hong Yan[1,2,7], Lei Bi[2,3,7], Yunshan Wang[2,4,7], Xia Zhang[5], Zhibo Hou[6], Qian Wang[1], Antoine M Snijders[2], Jian-Hua Mao[2]*

[1]Department of Laboratory Medicine, Nanjing Chest Hospital, 215 Guangzhou Road, Nanjing 210029, China.

[2]Biological Systems and Engineering Division, Lawrence Berkeley National Laboratory, Berkeley, California, USA.

[3]Department of Preclinical Medicine, Nanjing University of Chinese Medicine, 138 Xianlin Road, Nanjing 210023, China

[4]International Biotechnology R&D Center, Shandong University School of Ocean, Weihai, Shandong 264209, China.

[5]Department of Tuberculosis, Nanjing Chest Hospital, 215 Guangzhou Road, Nanjing 210029, China.

[6]Department of First Respiratory, Nanjing Chest Hospital, 215 Guangzhou Road, Nanjing 210029, China.

[7]These authors contributed equally.

*Correspondence to: Jian-Hua Mao: JHMao@lbl.gov

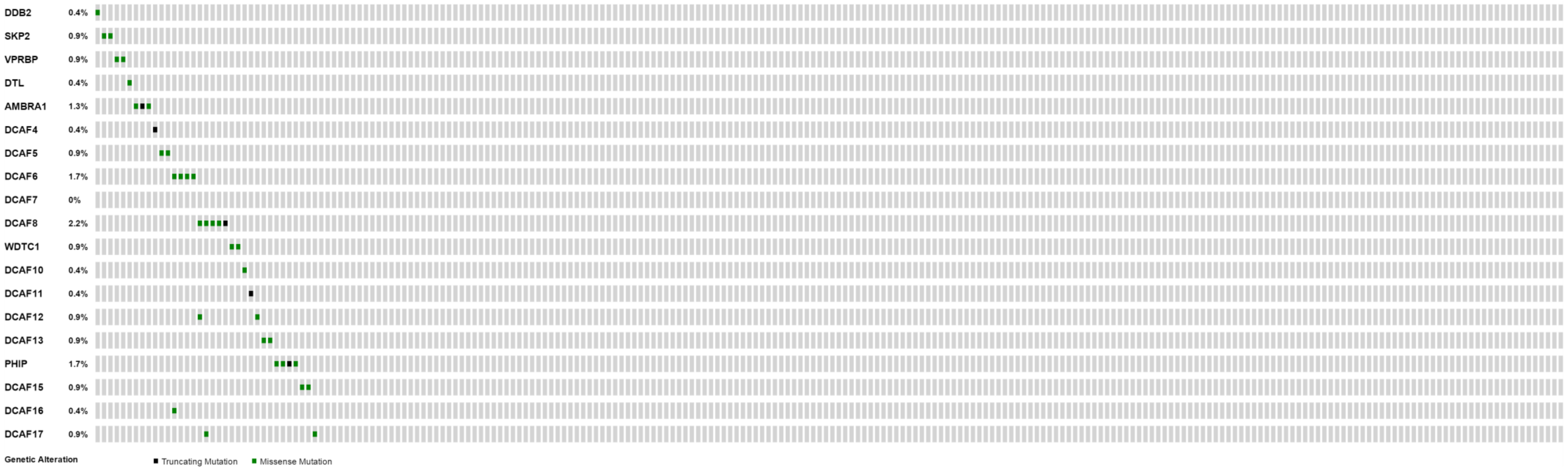

Figure S1. Mutation frequency of DCAFs in lung adenocarcinomas

Table S1. The p-values and fold change (FC) for DCAFs between LuADCs and normal lung tissues. Bold cases reached the following criteria: adjusted p-value <0.05 and fold change > 2 (logFC > 0.3).

Gene	Affymetrix probe	GSE19188			GSE31210			GSE19804		
		Adjusted p-Value	p-Value	logFC	Adjusted p-Value	p-Value	logFC	Adjusted p-Value	p-Value	logFC
DDB2	203409_at	**2.70E-04**	**6.85E-05**	**-0.378**	8.72E-01	7.97E-01	-0.028	7.40E-02	3.20E-02	0.206
SKP2	203626_s_at	1.23E-03	3.71E-04	0.256	6.27E-01	4.83E-01	0.176	2.93E-01	1.79E-01	0.131
	210567_s_at	2.28E-03	7.48E-04	0.289	9.04E-01	8.46E-01	0.023	5.59E-01	4.25E-01	0.093
	203625_x_at	**1.77E-07**	**2.24E-08**	**0.821**	7.47E-02	2.97E-02	0.309	**8.35E-05**	**1.32E-05**	**0.621**
VPRBP (DCAF1)	204376_at	**2.16E-13**	**7.97E-15**	**-0.697**	**1.37E-04**	**1.88E-05**	**-0.684**	**1.98E-05**	**2.67E-06**	**-0.350**
	226476_s_at	6.37E-01	5.58E-01	0.037	8.38E-01	7.50E-01	0.030	5.91E-01	4.59E-01	0.044
	226477_at	5.88E-05	1.26E-05	0.285	7.78E-04	1.37E-04	0.253	4.27E-01	2.94E-01	0.063
	204377_s_at	**2.93E-11**	**1.71E-12**	**-0.505**	**6.43E-04**	**1.10E-04**	**-0.515**	1.89E-03	4.38E-04	-0.250
	226481_at	6.34E-01	5.56E-01	0.036	7.99E-03	2.05E-03	0.184	7.95E-01	7.01E-01	0.023
DTL (DCAF2)	218585_s_at	**1.65E-11**	**9.18E-13**	**1.736**	1.60E-01	7.67E-02	0.399	**1.73E-02**	**5.68E-03**	**0.571**
	222680_s_at	**2.60E-14**	**7.80E-16**	**1.627**	**1.91E-02**	**5.74E-03**	**0.606**	**1.02E-02**	**3.06E-03**	**0.572**
AMBRA1 (DCAF3)	234237_s_at	1.39E-02	5.97E-03	0.075	3.73E-01	2.33E-01	0.340	8.85E-01	8.22E-01	-0.014
	219141_s_at	5.86E-01	5.04E-01	0.031	2.48E-01	1.35E-01	0.114	1.98E-05	2.67E-06	0.205
	52731_at	9.05E-01	8.74E-01	-0.012	1.06E-01	4.60E-02	0.125	2.04E-04	3.56E-05	0.229
DCAF4	214758_at	1.22E-01	7.55E-02	0.098	7.00E-01	5.69E-01	-0.072	5.13E-01	3.78E-01	0.037
	226938_at	**1.63E-04**	**3.89E-05**	**0.357**	**7.18E-03**	**1.81E-03**	**0.364**	**1.30E-05**	**1.67E-06**	**0.370**
DCAF5	1554558_at	5.80E-02	3.14E-02	0.095	7.87E-01	6.79E-01	-0.093	3.74E-01	2.47E-01	-0.035
	224696_s_at	2.18E-03	7.11E-04	-0.158	4.03E-02	1.40E-02	-0.252	6.47E-01	5.21E-01	0.043
	224697_at	3.85E-03	1.35E-03	-0.201	5.26E-01	3.76E-01	-0.088	9.63E-01	9.40E-01	-0.005
	224703_at	4.34E-02	2.24E-02	-0.127	6.42E-01	5.00E-01	0.055	1.03E-01	4.80E-02	0.180
	227251_at	**2.77E-08**	**2.99E-09**	**-0.408**	3.81E-01	2.40E-01	-0.103	5.04E-02	2.02E-02	-0.168
DCAF6	217908_s_at	8.97E-02	5.26E-02	-0.130	4.10E-01	2.66E-01	0.099	8.58E-01	7.86E-01	0.018
	232349_x_at	1.13E-01	6.92E-02	0.106	1.72E-01	8.45E-02	0.174	3.62E-01	2.37E-01	0.090
DCAF7	224748_at	2.15E-01	1.49E-01	0.097	**4.14E-07**	**2.56E-08**	**0.391**	1.52E-02	4.86E-03	0.182
	209592_s_at	**1.05E-03**	3.10E-04	0.224	9.70E-01	9.51E-01	0.005	7.91E-01	6.95E-01	-0.022
	221745_at	**9.27E-06**	**1.66E-06**	**0.535**	**1.65E-03**	**3.27E-04**	**0.309**	2.26E-01	1.29E-01	0.164
	224730_at	**1.96E-03**	**6.29E-04**	**0.408**	4.19E-01	2.73E-01	0.080	4.01E-01	2.70E-01	-0.137
	221744_at	4.44E-01	3.58E-01	0.071	8.03E-01	7.01E-01	0.025	4.41E-01	3.07E-01	0.079

DCAF8	202250_s_at	**5.32E-06**	**9.06E-07**	**-0.428**	5.92E-01	4.45E-01	-0.087	9.31E-01	8.89E-01	0.012
	202249_s_at	9.36E-01	9.13E-01	-0.004	1.12E-01	4.93E-02	0.206	4.21E-02	1.63E-02	0.145
	216885_s_at	1.67E-02	7.37E-03	-0.152	9.28E-01	8.82E-01	-0.015	4.44E-01	3.10E-01	0.062
	1554049_s_at	4.35E-02	2.24E-02	-0.129	6.41E-01	4.99E-01	-0.075	2.84E-01	1.73E-01	-0.071
WDTC1 (DCAF9)	216036_x_at	4.10E-01	3.25E-01	0.065	4.02E-01	2.59E-01	0.101	2.59E-01	1.53E-01	0.093
	40829_at	2.07E-02	9.46E-03	-0.160	7.53E-02	3.00E-02	0.121	5.30E-01	3.95E-01	0.058
	215497_s_at	3.95E-01	3.10E-01	-0.063	7.45E-01	6.26E-01	0.033	1.30E-02	4.07E-03	0.129
DCAF10	219001_s_at	3.65E-03	1.28E-03	-0.292	**1.14E-03**	**2.13E-04**	**-0.320**	9.34E-02	4.26E-02	-0.179
	226511_at	8.93E-01	8.59E-01	-0.014	**5.95E-04**	**1.01E-04**	**-0.334**	3.44E-01	2.21E-01	-0.115
	230679_at	7.24E-01	6.56E-01	0.043	6.86E-03	1.72E-03	-0.252	3.18E-01	2.00E-01	-0.121
	222804_x_at	**6.55E-04**	**1.84E-04**	**0.336**	**1.04E-03**	**1.91E-04**	**0.363**	8.91E-01	8.31E-01	0.024
DCAF11	201886_at	7.39E-01	6.73E-01	0.025	3.99E-01	2.56E-01	0.112	5.45E-03	1.49E-03	0.230
	216389_s_at	1.97E-03	6.33E-04	0.254	**4.08E-03**	**9.38E-04**	**0.528**	1.31E-01	6.44E-02	0.189
DCAF12	224789_at	**2.10E-06**	**3.29E-07**	**0.378**	**4.14E-06**	**3.51E-07**	**0.619**	**2.25E-10**	**9.84E-12**	**0.628**
DCAF13	225676_s_at	**1.60E-13**	**5.71E-15**	**0.940**	**9.51E-06**	**9.04E-07**	**0.546**	**2.01E-07**	**1.66E-08**	**0.566**
	220843_s_at	2.49E-03	8.28E-04	0.212	7.15E-01	5.88E-01	0.082	3.46E-01	2.23E-01	0.048
	231784_s_at	**1.50E-09**	**1.24E-10**	**0.868**	**5.82E-04**	**9.81E-05**	**0.483**	**1.25E-03**	**2.71E-04**	**0.543**
PHIP (DCAF14)	244811_at	9.89E-01	9.85E-01	0.002	**8.20E-03**	**2.11E-03**	**0.317**	7.00E-01	5.83E-01	-0.073
	212542_s_at	1.15E-01	7.07E-02	-0.145	4.91E-01	3.40E-01	-0.094	9.43E-01	9.09E-01	0.011
	213074_at	4.18E-01	3.33E-01	-0.061	5.02E-01	3.51E-01	0.080	2.10E-01	1.17E-01	0.138
DCAF15	221849_s_at	1.26E-01	7.89E-02	0.160	6.77E-01	5.41E-01	0.081	1.99E-01	1.09E-01	0.127
	221851_at	2.36E-02	1.10E-02	0.187	6.32E-01	4.88E-01	0.225	9.31E-01	8.90E-01	0.015
	91952_at	2.30E-01	1.61E-01	0.128	9.94E-01	9.90E-01	-0.001	4.05E-01	2.74E-01	0.074
DCAF16	239187_at	2.92E-01	2.15E-01	0.043	9.48E-01	9.16E-01	-0.028	1.62E-01	8.42E-02	0.062
	219717_at	**3.30E-05**	**6.70E-06**	**0.467**	**4.38E-03**	**1.02E-03**	**0.312**	7.38E-01	6.29E-01	0.045
	228106_at	5.96E-02	3.24E-02	0.178	5.61E-01	4.12E-01	-0.073	8.16E-01	7.28E-01	-0.028
DCAF17	231921_at	2.33E-01	1.64E-01	0.147	**4.29E-04**	**6.92E-05**	**0.381**	9.01E-01	8.46E-01	-0.022
	220172_at	1.84E-04	4.47E-05	0.274	**2.66E-08**	**1.14E-09**	**0.754**	3.24E-01	2.05E-01	0.135

Table S2. The impact of DCAFs on overall survival. Genes highlighted in red indicate consistent significance was observed across independent probe sets for a specific gene.

Gene	K-M Plotter analysis of microarray data			Cox regression analysis of TCGA data	
	Affymetrix probe	HR (95% CI)	p-value	HR (95% CI)	p-value
DDB2	203409_at	**0.70 (0.55-0.90)**	**4.40E-03**	0.96 (0.84-1.11)	0.60
SKP2	203626_s_at	**1.87 (1.48-2.36)**	**1.00E-07**	1.03 (0.99-1.07)	0.21
	210567_s_at	**1.64 (1.29-2.08)**	**4.20E-05**		
	203625_x_at	**0.69 (0.55-0.87)**	**1.70E-03**		
VPRBP	204376_at	**0.70 (0.55-0.89)**	**3.40E-03**	0.92 (0.81-1.05)	0.22
	226476_s_at	**1.86 (1.46-2.36)**	**2.90E-07**		
	226477_at	**1.56 (1.22-2.00)**	**3.40E-04**		
	204377_s_at	**0.53 (0.42-0.67)**	**1.20E-07**		
	226481_at	**0.39 (0.29-0.52)**	**8.20E-11**		
DTL	218585_s_at	**1.65 (1.28-2.13)**	**1.10E-04**	**1.25 (1.12-1.40)**	**0.000052**
	222680_s_at	**2.36 (1.80-3.08)**	**1.00E-10**		
AMBRA1	234237_s_at	**1.35 (1.05-1.73)**	**1.80E-02**	0.93 (0.81-1.05)	0.25
	219141_s_at	**0.47 (0.37-0.60)**	**6.10E-10**		
	52731_at	**0.47 (0.37-0.59)**	**8.80E-11**		
DCAF4	214758_at	**0.65 (0.52-0.82)**	**3.10E-04**	0.94 (0.83-1.06)	0.32
	226938_at	**0.53 (0.42-0.68)**	**2.30E-07**		
DCAF5	1554558_at	**1.56 (1.22-2.00)**	**3.30E-04**	0.93 (0.84-1.03)	0.15
	224696_s_at	**0.35 (0.25-0.50)**	**4.70E-10**		
	224697_at	0.81 (0.62-1.06)	1.20E-01		
	224703_at	**0.72 (0.56-0.93)**	**9.80E-03**		
	227251_at	0.77 (0.59-1.00)	5.40E-02		
DCAF6	217908_s_at	**0.34 (0.25-0.46)**	**7.70E-14**	0.95 (0.86-1.05)	0.29
	232349_x_at	1.24 (0.97-1.58)	8.50E-02		
DCAF7	224748_at	**1.35 (1.05-1.75)**	**2.10E-02**	0.97 (0.86-1.09)	0.61
	209592_s_at	**2.14 (1.69-2.70)**	**5.40E-11**		
	221745_at	**1.73 (1.35-2.23)**	**1.60E-05**		
	224730_at	**0.68 (0.53-0.87)**	**1.80E-03**		
	221744_at	**1.90 (1.50-2.42)**	**1.00E-07**		
DCAF8	202250_s_at	**0.61 (0.47-0.78)**	**8.10E-05**	0.96 (0.88-1.06)	0.42
	202249_s_at	**1.54 (1.18-2.01)**	**1.40E-03**		
	216885_s_at	**1.54 (1.22-1.94)**	**2.40E-04**		
	1554049_s_at	**0.37 (0.29-0.47)**	**<1.00E-16**		
WDTC1	216036_x_at	**1.69 (1.34-2.14)**	**8.00E-06**	**0.83 (0.73-0.94)**	**0.004**
	40829_at	**1.89 (1.46-2.44)**	**6.70E-07**		
	215497_s_at	**0.58 (0.46-0.74)**	**7.10E-06**		
DCAF10	219001_s_at	**1.58 (1.25-2.00)**	**1.30E-04**	1.02 (0.90-1.15)	0.76
	226511_at	**0.45 (0.34-0.60)**	**2.40E-08**		
	230679_at	**0.33 (0.23-0.49)**	**3.00E-09**		
	222804_x_at	1.22 (0.96-1.55)	1.10E-01		

DCAF11	201886_at	**0.73 (0.57-0.93)**	**1.20E-02**	**0.88 (0.79-0.99)**	**0.035**
	216389_s_at	**2.96 (2.17-4.03)**	**4.90E-13**		
DCAF12	224789_at	**0.60 (0.47-0.77)**	**3.60E-05**	0.93 (0.81-1.08)	0.34
DCAF13	225676_s_at	0.79 (0.62-1.01)	6.10E-02	1.05 (0.98-1.12)	0.16
	220843_s_at	**0.62 (0.49-0.78)**	**5.10E-05**		
	231784_s_at	**0.58 (0.45-0.74)**	**6.90E-06**		
PHIP	244811_at	**0.27 (0.18-0.39)**	**7.60E-14**	0.87 (0.75-1.02)	0.081
	212542_s_at	**1.40 (1.10-1.79)**	**6.60E-03**		
	213074_at	**0.39 (0.30-0.50)**	**8.40E-15**		
DCAF15	221849_s_at	**2.37 (1.87-3.01)**	**2.20E-13**	0.91 (0.79-1.05)	0.19
	221851_at	**2.52 (1.98-3.21)**	**1.10E-14**		
	91952_at	**2.63 (2.08-3.33)**	**<1.00E-16**		
DCAF16	239187_at	**1.84 (1.45-2.34)**	**4.60E-07**	0.94 (0.84-1.06)	0.34
	219717_at	**0.60 (0.47-0.77)**	**3.00E-05**		
	228106_at	**1.53 (1.19-1.96)**	**8.10E-04**		
DCAF17	231921_at	**0.61 (0.48-0.77)**	**3.80E-05**	1.04 (0.94-1.16)	0.43
	220172_at	0.79 (0.62-1.01)	5.60E-02		

Table S3. **List of genes that are co-expressed with DTL**

Gene	Spearman correlation coefficent (R)
EXO1	0.89
NEK2	0.85
KIF14	0.85
CDC6	0.84
KIF11	0.84
ORC1	0.84
BRCA1	0.83
CENPF	0.83
ASPM	0.83
MCM10	0.83
KIF4A	0.83
TPX2	0.83
CKAP2L	0.83
BUB1B	0.82
BUB1	0.82
CDCA5	0.82
KIF23	0.82
ZWINT	0.82
PLK4	0.82
FANCI	0.82
NCAPH	0.82
SGOL1	0.82
NCAPG	0.82
CLSPN	0.82
CENPI	0.81
MCM6	0.81
STIL	0.81
RRM2	0.81
TOP2A	0.81
PRC1	0.81
DLGAP5	0.81
ARHGAP11A	0.81
DIAPH3	0.81
KIF15	0.81
DEPDC1	0.81
BRIP1	0.81
CCNA2	0.80
CHEK1	0.80
CENPE	0.80
FAM72A	0.80
HELLS	0.80
KIFC1	0.80
PLK1	0.80
TTK	0.80
MELK	0.80

KIF2C	0.80
WDHD1	0.80
NUSAP1	0.80
ANLN	0.80
FAM72B	0.80
TICRR	0.80
RAD54L	0.79
SHCBP1	0.79
WDR76	0.79
NDC80	0.79
SPAG5	0.79
FAM72D	0.79
CDCA8	0.79
SKA3	0.79
RACGAP1	0.79
CDK1	0.78
CENPA	0.78
FEN1	0.78
FOXM1	0.78
LMNB1	0.78
MCM4	0.78
MCM2	0.78
MKI67	0.78
KIF18B	0.78
CCNB2	0.78
ESPL1	0.78
CDT1	0.78
CDCA2	0.78
GTSE1	0.78
CASC5	0.78
HJURP	0.78
PRR11	0.78
ERCC6L	0.78
SKA1	0.78
KIAA1524	0.78
UHRF1	0.78
CDC25C	0.77
XRCC2	0.77
KIF20A	0.77
NUF2	0.77
ZNF367	0.77
NCAPG2	0.77
CEP55	0.77
SPC24	0.77
FAM111B	0.77
SPC25	0.77
BLM	0.76
CCNB1	0.76
CDC25A	0.76

CDC20	0.76
HMMR	0.76
KPNA2	0.76
MAD2L1	0.76
MYBL2	0.76
RAD51	0.76
TYMS	0.76
CDC45	0.76
KIF20B	0.76
POLQ	0.76
ESCO2	0.76
ASF1B	0.76
IQGAP3	0.76
SGOL2	0.76
AUNIP	0.75
RAD51AP1	0.75
DEPDC1B	0.75
UBE2T	0.75
MTFR2	0.74
PKMYT1	0.74
GINS1	0.74
KIF18A	0.74
DDIAS	0.74
ATAD2	0.74
CDKN3	0.73
EME1	0.73
DSCC1	0.73
CENPU	0.73
SMC2	0.73
GSG2	0.73
GINS4	0.73
BIRC5	0.72
FANCB	0.72
AURKA	0.72
TIMELESS	0.72
AURKB	0.72
CDC7	0.72
CHAF1B	0.72
CENPO	0.72
CHAF1A	0.72
TROAP	0.72
ATAD5	0.72
FAM64A	0.72
ORC6	0.72
CENPL	0.72
CENPH	0.72
CENPK	0.72
E2F2	0.71
INCENP	0.71

E2F7	0.71
RFC4	0.71
TCF19	0.71
KIAA0101	0.71
C17ORF53	0.71
TRIP13	0.71
DBF4	0.71
OIP5	0.71
ARHGAP11B	0.71
C1ORF112	0.71
PARPBP	0.71
E2F1	0.70
FANCD2	0.70
POLE2	0.70
E2F8	0.70
TACC3	0.70
CDCA3	0.70
PBK	0.70
BRCA2	0.69
CDK2	0.69
ECT2	0.69
MCM7	0.69
KIF4B	0.69
TOPBP1	0.69
UBE2C	0.69
GINS2	0.69
CENPN	0.69
CCNE1	0.68
EZH2	0.68
MCM3	0.68
RBL1	0.68
TK1	0.68
GMPS	0.68
CCNE2	0.68
KNTC1	0.68
FAM83D	0.68
CENPW	0.68
SASS6	0.68
NUP205	0.68
FBXO5	0.68
CKAP2	0.68
POC1A	0.68
C5ORF34	0.68
GINS3	0.68
CCNF	0.67
MSH6	0.67
RFC3	0.67
RRM1	0.67
TRAIP	0.67

ZWILCH	0.67
MASTL	0.67
MND1	0.67
DNAJC9	0.67
MTBP	0.67
PRIM1	0.66
RFC5	0.66
TMPO	0.66
SPDL1	0.66
NEIL3	0.66
MIS18A	0.66
DONSON	0.66
DNA2	0.65
KPNB1	0.65
MSH2	0.65
VRK1	0.65
NCAPD2	0.65
FBXO45	0.65
DSN1	0.65
NDC1	0.65
POLA2	0.65
LMNB2	0.65
TBC1D31	0.65
SUV39H1	0.64
WDR62	0.64
SAPCD2	0.64
R3HDM1	0.64
PSMC3IP	0.64
CKS1B	0.63
FANCG	0.63
SKP2	0.63
TMPO-AS1	0.63
NUP155	0.63
C16ORF59	0.63
MCM8	0.63
PSRC1	0.63
MMS22L	0.63
CSE1L	0.62
H2AFZ	0.62
CENPM	0.62
TUBA1B	0.62
RNASEH2A	0.62
LIN9	0.62
RFWD3	0.62
NCAPD3	0.62
RAD54B	0.62
CTSV	0.61
HMGB2	0.61
PCNA	0.61

UNG	0.61
C18ORF54	0.61
CDCA4	0.61
RMI2	0.61
KNSTRN	0.61
FANCA	0.60
GEN1	0.60
PPM1G	0.60
PPAT	0.60
SUV39H2	0.60
TIPIN	0.60
ARHGEF39	0.60
NEMP1	0.60
CST3	-0.60
CRY2	-0.64
C16ORF89	-0.64

Table S4. Gene ontology and KEGG analysis of DTL co-expressed genes

Category	Term		Fold Enrichment	P-Value	Bonferroni	Benjamini	FDR
GOTERM_BP_FAT	GO:0007049	cell cycle	11.13	8.57E-110	7.68E-107	7.68E-107	1.34E-106
GOTERM_BP_FAT	GO:0022403	cell cycle phase	15.69	4.49E-93	4.03E-90	2.01E-90	7.00E-90
GOTERM_BP_FAT	GO:0000279	M phase	17.91	6.72E-89	6.03E-86	2.01E-86	1.05E-85
GOTERM_BP_FAT	GO:0022402	cell cycle process	12.21	1.00E-87	8.98E-85	2.25E-85	1.56E-84
GOTERM_BP_FAT	GO:0000278	mitotic cell cycle	14.84	7.23E-75	6.48E-72	1.30E-72	1.13E-71
GOTERM_BP_FAT	GO:0000280	nuclear division	20.70	1.01E-71	9.06E-69	1.51E-69	1.58E-68
GOTERM_BP_FAT	GO:0007067	mitosis	20.70	1.01E-71	9.06E-69	1.51E-69	1.58E-68
GOTERM_BP_FAT	GO:0000087	M phase of mitotic cell cycle	20.33	4.07E-71	3.65E-68	5.21E-69	6.34E-68
GOTERM_BP_FAT	GO:0048285	organelle fission	19.89	2.22E-70	1.99E-67	2.49E-68	3.47E-67
GOTERM_BP_FAT	GO:0051301	cell division	15.66	1.26E-63	1.13E-60	1.25E-61	1.96E-60
GOTERM_BP_FAT	GO:0006259	DNA metabolic process	9.93	2.73E-54	2.45E-51	2.45E-52	4.25E-51
GOTERM_BP_FAT	GO:0006260	DNA replication	17.27	2.45E-46	2.19E-43	1.99E-44	3.81E-43
GOTERM_BP_FAT	GO:0007059	chromosome segregation	26.46	2.37E-36	2.13E-33	1.77E-34	3.70E-33
GOTERM_BP_FAT	GO:0051726	regulation of cell cycle	9.71	4.72E-33	4.23E-30	3.25E-31	7.35E-30
GOTERM_BP_FAT	GO:0006974	response to DNA damage stimulus	8.62	1.18E-30	1.06E-27	7.58E-29	1.84E-27
GOTERM_BP_FAT	GO:0006281	DNA repair	9.67	6.03E-28	5.40E-25	3.60E-26	9.39E-25
GOTERM_BP_FAT	GO:0033554	cellular response to stress	5.68	1.13E-22	1.01E-19	6.32E-21	1.76E-19
GOTERM_BP_FAT	GO:0000075	cell cycle checkpoint	17.66	2.12E-22	1.90E-19	1.12E-20	3.30E-19
GOTERM_BP_FAT	GO:0007017	microtubule-based process	9.00	4.94E-22	4.42E-19	2.46E-20	7.69E-19
GOTERM_BP_FAT	GO:0007346	regulation of mitotic cell cycle	12.34	9.09E-22	8.15E-19	4.29E-20	1.42E-18
GOTERM_BP_FAT	GO:0010564	regulation of cell cycle process	14.69	2.74E-21	2.46E-18	1.23E-19	4.27E-18
GOTERM_BP_FAT	GO:0051276	chromosome organization	5.94	6.11E-21	5.47E-18	2.61E-19	9.51E-18
GOTERM_BP_FAT	GO:0000070	mitotic sister chromatid segregation	31.62	1.71E-20	1.53E-17	6.96E-19	2.66E-17
GOTERM_BP_FAT	GO:0000819	sister chromatid segregation	30.77	2.97E-20	2.66E-17	1.16E-18	4.63E-17
GOTERM_BP_FAT	GO:0006261	DNA-dependent DNA replication	20.78	4.75E-18	4.26E-15	1.77E-16	7.40E-15
GOTERM_BP_FAT	GO:0007126	meiosis	14.35	1.25E-17	1.12E-14	4.48E-16	1.95E-14
GOTERM_BP_FAT	GO:0051327	M phase of meiotic cell cycle	14.35	1.25E-17	1.12E-14	4.48E-16	1.95E-14
GOTERM_BP_FAT	GO:0051321	meiotic cell cycle	14.06	1.91E-17	1.71E-14	6.57E-16	2.97E-14
GOTERM_BP_FAT	GO:0000226	microtubule cytoskeleton	10.93	2.11E-17	1.89E-14	7.00E-16	3.29E-14
GOTERM_BP_FAT	GO:0051329	interphase of mitotic cell cycle	12.35	1.05E-14	9.45E-12	3.38E-13	1.64E-11
GOTERM_BP_FAT	GO:0051325	interphase	12.00	1.78E-14	1.59E-11	5.49E-13	2.77E-11

GOTERM_BP_FAT	GO:0007051	spindle organization	20.84	5.60E-14	5.02E-11	1.67E-12	8.73E-11
GOTERM_BP_FAT	GO:0006323	DNA packaging	10.30	1.38E-12	1.24E-09	3.99E-11	2.15E-09
GOTERM_BP_FAT	GO:0006310	DNA recombination	10.84	3.04E-12	2.72E-09	8.51E-11	4.74E-09
GOTERM_BP_FAT	GO:0007093	mitotic cell cycle checkpoint	18.69	2.25E-11	2.02E-08	6.11E-10	3.50E-08
GOTERM_BP_FAT	GO:0031570	DNA integrity checkpoint	15.45	2.10E-10	1.88E-07	5.54E-09	3.27E-07
GOTERM_BP_FAT	GO:0008283	cell proliferation	4.30	3.07E-10	2.75E-07	7.85E-09	4.78E-07
GOTERM_BP_FAT	GO:0051783	regulation of nuclear division	14.35	4.92E-10	4.41E-07	1.22E-08	7.66E-07
GOTERM_BP_FAT	GO:0007088	regulation of mitosis	14.35	4.92E-10	4.41E-07	1.22E-08	7.66E-07
GOTERM_BP_FAT	GO:0007010	cytoskeleton organization	4.15	1.51E-09	1.35E-06	3.65E-08	2.35E-06
GOTERM_BP_FAT	GO:0006302	double-strand break repair	12.96	1.55E-09	1.39E-06	3.66E-08	2.42E-06
GOTERM_BP_FAT	GO:0030261	chromosome condensation	24.11	1.80E-09	1.61E-06	4.13E-08	2.80E-06
GOTERM_BP_FAT	GO:0000724	double-strand break repair via homologous recombination	28.20	6.24E-09	5.59E-06	1.40E-07	9.72E-06
GOTERM_BP_FAT	GO:0000725	recombinational repair	28.20	6.24E-09	5.59E-06	1.40E-07	9.72E-06
GOTERM_BP_FAT	GO:0000910	cytokinesis	16.33	6.85E-09	6.14E-06	1.50E-07	1.07E-05
GOTERM_BP_FAT	GO:0051052	regulation of DNA metabolic process	8.22	1.36E-08	1.22E-05	2.89E-07	2.11E-05
GOTERM_BP_FAT	GO:0007076	mitotic chromosome condensation	36.06	1.57E-08	1.41E-05	3.27E-07	2.45E-05
GOTERM_BP_FAT	GO:0048015	phosphoinositide-mediated signaling	9.13	6.99E-08	6.26E-05	1.42E-06	1.09E-04
GOTERM_BP_FAT	GO:0006270	DNA replication initiation	29.30	7.06E-08	6.33E-05	1.41E-06	1.10E-04
GOTERM_BP_FAT	GO:0007018	microtubule-based movement	7.70	1.11E-07	9.93E-05	2.16E-06	1.73E-04
GOTERM_BP_FAT	GO:0051640	organelle localization	8.74	1.11E-07	9.99E-05	2.12E-06	1.74E-04
GOTERM_BP_FAT	GO:0033043	regulation of organelle organization	5.25	1.48E-07	1.33E-04	2.77E-06	2.31E-04
GOTERM_BP_FAT	GO:0042770	DNA damage response, signal transduction	9.21	2.80E-07	2.50E-04	5.11E-06	4.35E-04
GOTERM_BP_FAT	GO:0000077	DNA damage checkpoint	12.56	4.68E-07	4.20E-04	8.39E-06	7.30E-04
GOTERM_BP_FAT	GO:0031577	spindle checkpoint	33.49	5.01E-07	4.49E-04	8.81E-06	7.81E-04
GOTERM_BP_FAT	GO:0030071	regulation of mitotic metaphase/anaphase transition	21.31	6.11E-07	5.47E-04	1.05E-05	9.52E-04
GOTERM_BP_FAT	GO:0051656	establishment of organelle	9.71	7.74E-07	6.93E-04	1.31E-05	0.001206
GOTERM_BP_FAT	GO:0065004	protein-DNA complex assembly	8.10	9.47E-07	8.48E-04	1.57E-05	0.001475
GOTERM_BP_FAT	GO:0000079	regulation of cyclin-dependent protein kinase activity	11.16	1.20E-06	0.001071756	1.95E-05	0.001864
GOTERM_BP_FAT	GO:0006268	DNA unwinding during replication	28.70	1.24E-06	0.00110778	1.98E-05	0.001927

GOTERM_BP_FAT	GO:0010948	negative regulation of cell cycle process	18.75	1.40E-06	0.001251283	2.20E-05	0.002177
GOTERM_BP_FAT	GO:0051053	negative regulation of DNA metabolic process	13.74	1.48E-06	0.00132541	2.29E-05	0.002306
GOTERM_BP_FAT	GO:0034621	cellular macromolecular complex subunit organization	3.75	1.56E-06	0.001395403	2.37E-05	0.002428
GOTERM_BP_FAT	GO:0051303	establishment of chromosome localization	26.79	1.83E-06	0.001641215	2.74E-05	0.002856
GOTERM_BP_FAT	GO:0050000	chromosome localization	26.79	1.83E-06	0.001641215	2.74E-05	0.002856
GOTERM_BP_FAT	GO:0007052	mitotic spindle organization	26.79	1.83E-06	0.001641215	2.74E-05	0.002856
GOTERM_BP_FAT	GO:0010212	response to ionizing radiation	10.05	2.73E-06	0.002440444	4.01E-05	0.004248
GOTERM_BP_FAT	GO:0006275	regulation of DNA replication	9.72	3.51E-06	0.003142947	5.08E-05	0.005473
GOTERM_BP_FAT	GO:0032508	DNA duplex unwinding	22.32	5.04E-06	0.004509046	7.17E-05	0.007857
GOTERM_BP_FAT	GO:0032392	DNA geometric change	22.32	5.04E-06	0.004509046	7.17E-05	0.007857
GOTERM_BP_FAT	GO:0007094	mitotic cell cycle spindle assembly checkpoint	30.44	1.44E-05	0.012806877	2.01E-04	0.022408
GOTERM_BP_FAT	GO:0045841	negative regulation of mitotic metaphase/anaphase transition	30.44	1.44E-05	0.012806877	2.01E-04	0.022408
GOTERM_BP_FAT	GO:0043933	macromolecular complex subunit organization	2.55	1.90E-05	0.016870837	2.62E-04	0.029579
GOTERM_BP_FAT	GO:0006333	chromatin assembly or disassembly	5.80	1.94E-05	0.017227913	2.63E-04	0.03021
GOTERM_BP_FAT	GO:0051784	negative regulation of nuclear division	27.90	2.13E-05	0.018928393	2.85E-04	0.03322
GOTERM_BP_FAT	GO:0045839	negative regulation of mitosis	27.90	2.13E-05	0.018928393	2.85E-04	0.03322
GOTERM_BP_FAT	GO:0034622	cellular macromolecular complex assembly	3.58	2.16E-05	0.0192039	2.85E-04	0.033708
GOTERM_BP_FAT	GO:0006271	DNA strand elongation during DNA replication	53.58	3.16E-05	0.027929722	4.10E-04	0.04924
GOTERM_CC_FAT	GO:0005694	chromosome	12.00	8.00E-60	1.35E-57	1.35E-57	9.75E-57
GOTERM_CC_FAT	GO:0000793	condensed chromosome	26.97	1.99E-53	3.36E-51	1.68E-51	2.42E-50
GOTERM_CC_FAT	GO:0044427	chromosomal part	12.54	7.46E-53	1.26E-50	4.20E-51	9.09E-50
GOTERM_CC_FAT	GO:0000775	chromosome, centromeric region	24.40	4.12E-44	6.97E-42	1.74E-42	5.02E-41
GOTERM_CC_FAT	GO:0043228	non-membrane-bounded organelle	3.41	6.63E-43	1.12E-40	2.24E-41	8.08E-40
GOTERM_CC_FAT	GO:0043232	intracellular non-membrane-bounded organelle	3.41	6.63E-43	1.12E-40	2.24E-41	8.08E-40

GOTERM_CC_FAT	GO:0005819	spindle	19.55	9.02E-38	1.52E-35	2.54E-36	1.10E-34
GOTERM_CC_FAT	GO:0000779	condensed chromosome, centromeric region	33.23	4.49E-36	7.59E-34	1.08E-34	5.47E-33
GOTERM_CC_FAT	GO:0015630	microtubule cytoskeleton	7.85	3.65E-35	6.17E-33	7.71E-34	4.45E-32
GOTERM_CC_FAT	GO:0000777	condensed chromosome kinetochore	33.90	1.75E-32	2.95E-30	3.28E-31	2.13E-29
GOTERM_CC_FAT	GO:0005654	nucleoplasm	5.32	6.39E-29	1.08E-26	1.08E-27	7.79E-26
GOTERM_CC_FAT	GO:0000776	kinetochore	25.54	9.36E-29	1.58E-26	1.44E-27	1.14E-25
GOTERM_CC_FAT	GO:0031981	nuclear lumen	3.96	4.55E-28	7.68E-26	6.40E-27	5.54E-25
GOTERM_CC_FAT	GO:0044430	cytoskeletal part	4.69	1.58E-24	2.67E-22	2.06E-23	1.93E-21
GOTERM_CC_FAT	GO:0070013	intracellular organelle lumen	3.23	1.91E-22	3.24E-20	2.31E-21	2.33E-19
GOTERM_CC_FAT	GO:0043233	organelle lumen	3.16	7.73E-22	1.31E-19	8.71E-21	9.43E-19
GOTERM_CC_FAT	GO:0031974	membrane-enclosed lumen	3.10	2.55E-21	4.31E-19	2.69E-20	3.11E-18
GOTERM_CC_FAT	GO:0000228	nuclear chromosome	11.67	7.63E-19	1.29E-16	7.58E-18	9.29E-16
GOTERM_CC_FAT	GO:0005856	cytoskeleton	3.29	2.39E-17	4.04E-15	2.24E-16	2.91E-14
GOTERM_CC_FAT	GO:0005815	microtubule organizing center	7.47	2.58E-14	4.37E-12	2.30E-13	3.15E-11
GOTERM_CC_FAT	GO:0005874	microtubule	6.90	1.52E-13	2.56E-11	1.28E-12	1.85E-10
GOTERM_CC_FAT	GO:0005813	centrosome	7.43	1.52E-12	2.57E-10	1.22E-11	1.85E-09
GOTERM_CC_FAT	GO:0005876	spindle microtubule	28.69	1.90E-12	3.21E-10	1.46E-11	2.31E-09
GOTERM_CC_FAT	GO:0000922	spindle pole	22.25	3.74E-10	6.31E-08	2.75E-09	4.55E-07
GOTERM_CC_FAT	GO:0000794	condensed nuclear chromosome	16.98	6.21E-10	1.05E-07	4.38E-09	7.57E-07
GOTERM_CC_FAT	GO:0044454	nuclear chromosome part	9.30	6.66E-10	1.13E-07	4.50E-09	8.12E-07
GOTERM_CC_FAT	GO:0030496	midbody	33.61	1.68E-09	2.85E-07	1.09E-08	2.05E-06
GOTERM_CC_FAT	GO:0005657	replication fork	21.27	6.06E-09	1.02E-06	3.79E-08	7.39E-06
GOTERM_CC_FAT	GO:0000785	chromatin	6.05	5.77E-08	9.76E-06	3.48E-07	7.04E-05
GOTERM_CC_FAT	GO:0000796	condensin complex	63.03	4.23E-07	7.15E-05	2.47E-06	5.16E-04
GOTERM_CC_FAT	GO:0000940	outer kinetochore of condensed chromosome	42.02	3.45E-06	5.82E-04	1.94E-05	0.004201
GOTERM_CC_FAT	GO:0031262	Ndc80 complex	75.63	8.84E-06	0.001492236	4.82E-05	0.010769
GOTERM_MF_FAT	GO:0005524	ATP binding	3.54	1.47E-20	3.81E-18	3.81E-18	1.92E-17
GOTERM_MF_FAT	GO:0032559	adenyl ribonucleotide binding	3.49	2.96E-20	7.66E-18	3.83E-18	3.86E-17
GOTERM_MF_FAT	GO:0030554	adenyl nucleotide binding	3.31	4.32E-19	1.12E-16	3.73E-17	5.64E-16
GOTERM_MF_FAT	GO:0001883	purine nucleoside binding	3.26	9.33E-19	2.42E-16	6.04E-17	1.22E-15
GOTERM_MF_FAT	GO:0001882	nucleoside binding	3.24	1.32E-18	3.42E-16	6.84E-17	1.72E-15
GOTERM_MF_FAT	GO:0032555	purine ribonucleotide binding	2.89	3.10E-16	8.63E-14	1.44E-14	4.33E-13

GOTERM_MF_FAT	GO:0032553	ribonucleotide binding	2.89	3.10E-16	8.63E-14	1.44E-14	4.33E-13
GOTERM_MF_FAT	GO:0017076	purine nucleotide binding	2.77	2.26E-15	5.75E-13	8.22E-14	2.90E-12
GOTERM_MF_FAT	GO:0000166	nucleotide binding	2.44	3.30E-13	8.55E-11	1.07E-11	4.31E-10
GOTERM_MF_FAT	GO:0003677	DNA binding	2.17	6.38E-10	1.65E-07	1.83E-08	8.33E-07
GOTERM_MF_FAT	GO:0003777	microtubule motor activity	12.73	2.05E-09	5.30E-07	5.30E-08	2.67E-06
GOTERM_MF_FAT	GO:0008094	DNA-dependent ATPase activity	14.33	2.54E-08	6.59E-06	5.99E-07	3.32E-05
GOTERM_MF_FAT	GO:0003684	damaged DNA binding	14.70	1.40E-07	3.63E-05	3.03E-06	1.83E-04
GOTERM_MF_FAT	GO:0043566	structure-specific DNA binding	7.32	1.95E-07	5.04E-05	3.88E-06	2.54E-04
GOTERM_MF_FAT	GO:0003697	single-stranded DNA binding	13.36	3.02E-07	7.82E-05	5.59E-06	3.94E-04
GOTERM_MF_FAT	GO:0003774	motor activity	6.90	1.24E-06	3.21E-04	2.14E-05	0.001618
GOTERM_MF_FAT	GO:0003682	chromatin binding	6.53	2.13E-06	5.51E-04	3.44E-05	0.002779
GOTERM_MF_FAT	GO:0004386	helicase activity	6.42	7.93E-06	0.002050658	1.21E-04	0.010353
GOTERM_MF_FAT	GO:0003678	DNA helicase activity	14.29	8.05E-06	0.002083748	1.16E-04	0.01052
GOTERM_MF_FAT	GO:0004674	protein serine/threonine kinase activity	3.42	1.91E-05	0.004927651	2.60E-04	0.024912
KEGG_PATHWAY	hsa04110	Cell cycle	17.36	7.15E-32	2.57E-30	2.57E-30	6.38E-29
KEGG_PATHWAY	hsa03030	DNA replication	28.25	1.53E-17	5.51E-16	2.75E-16	1.36E-14
KEGG_PATHWAY	hsa04114	Oocyte meiosis	9.86	2.55E-11	9.19E-10	3.06E-10	2.28E-08
KEGG_PATHWAY	hsa04914	Progesterone-mediated oocyte maturation	8.67	3.35E-07	1.20E-05	3.01E-06	2.98E-04
KEGG_PATHWAY	hsa03430	Mismatch repair	20.63	6.42E-07	2.31E-05	4.62E-06	5.73E-04
KEGG_PATHWAY	hsa03440	Homologous recombination	16.95	2.26E-06	8.14E-05	1.36E-05	0.002017
KEGG_PATHWAY	hsa04115	p53 signaling pathway	8.97	5.03E-06	1.81E-04	2.59E-05	0.004485

Table S5. List of genes for which protein levels are altered between the LuADC group that overexpress DTL versus those without DTL overexpression

Gene	Mean protein expression		Standard deviation of protein expression		p-Value	q-Value
	DTL altered group (N=58)	DTL unaltered group (N=457)	DTL altered group (N=58)	DTL unaltered group (N=457)		
NAPSA	-0.15	0.17	0.2	0.65	2.00E-12	**3.90E-10**
CCNB1	1	-0.02	0.8	0.72	7.75E-11	**7.55E-09**
PCNA	0.29	0.01	0.32	0.26	7.62E-07	**4.95E-05**
TFRC	0.94	0.05	1.1	0.76	2.86E-06	**1.13E-04**
FOXM1	0.48	0.03	0.55	0.38	2.91E-06	**1.13E-04**
MSH6	0.46	-0.05	0.66	0.42	8.34E-06	**2.71E-04**
TGM2	-0.14	0.15	0.38	0.55	2.56E-05	**7.13E-04**
CCNE1	0.32	0.03	0.43	0.41	7.16E-05	**1.75E-03**
CCNE2	0.19	0.01	0.28	0.2	1.28E-04	**2.62E-03**
PRKAA1	-0.1	0.04	0.22	0.26	1.46E-04	**2.62E-03**
MSH2	0.27	-0.08	0.56	0.41	1.58E-04	**2.62E-03**
PDCD4	-0.47	0.06	0.85	0.62	1.63E-04	**2.62E-03**
PRKCD_PS664	-0.09	0.03	0.19	0.19	1.75E-04	**2.62E-03**
STMN1	0.26	0.04	0.4	0.25	8.65E-04	**0.0121**
ASNS	0.53	0.03	0.94	0.71	1.28E-03	**0.0166**
EIF4EBP1	0.32	0.05	0.52	0.48	1.57E-03	**0.0191**
BCL2L11	0.34	0	0.71	0.39	2.58E-03	**0.0296**
CAV1	-0.44	0.16	1.21	1.16	2.76E-03	**0.0299**
CASP7	0.54	0.13	0.86	0.69	3.08E-03	**0.0316**
SMAD1	0.16	0.01	0.33	0.19	4.09E-03	**0.039**
SERPINE1	0.77	0.15	1.32	1.07	4.34E-03	**0.039**
EIF4EBP1_PS65	0.13	0.01	0.24	0.22	4.40E-03	**0.039**
NKX2-1	-1.14	-0.15	2.19	1.33	4.78E-03	**0.0406**
PRKCA	-0.14	0.06	0.44	0.41	5.95E-03	**0.0483**
CD274	0.55	0.11	1.06	1.03	6.35E-03	**0.0495**
ADAR	0.04	0	0.1	0.14	6.88E-03	0.0516
ERBB3	-0.11	0.1	0.47	0.45	7.22E-03	0.0521
RPS6KA1	-0.11	0.08	0.42	0.4	7.74E-03	0.0539

KIT	-0.09	0.28	0.87	0.77	9.08E-03	0.0611
FN1	0.52	0.09	1.01	0.78	9.54E-03	0.062
PRKCB_PS660	-0.28	0.05	0.78	0.61	9.96E-03	0.0626
CDH1	-0.43	-0.06	0.92	0.71	0.0106	0.0641
PTEN	-0.21	-0.04	0.4	0.39	0.0108	0.0641
TYMS	0.16	-0.01	0.49	0.22	0.012	0.0689
FOXO3_PS318	-0.07	0.01	0.21	0.17	0.0134	0.0728
BECN1	-0.09	0	0.22	0.21	0.0134	0.0728
BAP1	0.08	-0.05	0.35	0.3	0.0139	0.0734
DIABLO	-0.02	0.15	0.4	0.58	0.0146	0.0739
EIF4EBP1_PT70	0.16	0.03	0.32	0.31	0.0148	0.0739
CHEK1	0.23	0.05	0.47	0.35	0.0173	0.0843
SETD2	0.02	0.23	0.51	0.79	0.0205	0.0977
CDKN2A	0.23	0.04	0.55	0.41	0.022	0.102
CDK1	0.06	-0.02	0.21	0.18	0.0237	0.107
SNAI1	0.05	0.29	0.59	1.05	0.0243	0.107
XRCC1	0.12	0.02	0.28	0.23	0.0274	0.118
KRT5	-0.06	0.02	0.23	0.38	0.0281	0.118
EZH2	0.11	0.04	0.21	0.21	0.0285	0.118
BAD_PS112	-0.13	-0.01	0.37	0.27	0.0342	0.138
ERCC1	-0.03	0.08	0.29	0.54	0.035	0.138
MET	0.05	0.26	0.54	0.96	0.0354	0.138
RAF1	0.1	0.03	0.21	0.17	0.0379	0.145
MYH11	-0.7	-0.07	1.94	1.37	0.0405	0.15
PDK1	-0.06	0.01	0.23	0.19	0.0406	0.15
RAD51	0.1	-0.01	0.34	0.28	0.0452	0.162
CTNNB1	-0.36	-0.08	0.84	0.76	0.0458	0.162
RAB25	-0.14	0.02	0.48	0.56	0.0478	0.166
CLDN7	-0.04	0.23	0.85	0.93	0.0522	0.179
MAPK14	-0.07	0.01	0.26	0.26	0.0537	0.18
MAPK8_PT183	-0.01	0.05	0.22	0.25	0.0579	0.189
ARAF_PS299	-0.04	0.11	0.48	0.41	0.0582	0.189
CASP3	0.12	0.03	0.31	0.32	0.0602	0.192
NFE2L2	0.1	0.04	0.24	0.23	0.0628	0.196
RB1	0.02	0.13	0.34	0.52	0.0632	0.196
SMAD3	0.09	0.03	0.21	0.17	0.0678	0.207

CDKN1B	-0.06	0.01	0.27	0.24	0.0737	0.221
CDKN1A	0.14	0	0.44	0.59	0.0769	0.227
GSK3B	0.05	-0.04	0.32	0.26	0.0794	0.228
GSK3A	0.05	-0.04	0.32	0.26	0.0794	0.228
CHGA	-0.05	0.01	0.26	0.24	0.0863	0.243
CASP8	0.05	0.2	0.5	0.74	0.0873	0.243
RBM15	0.1	-0.06	0.63	0.52	0.1	0.275
ACVRL1	-0.05	0.03	0.28	0.23	0.108	0.292
TP63	-0.1	0.1	0.91	0.9	0.111	0.297
PGR	0.01	0.08	0.27	0.24	0.115	0.302
SCD	-0.02	0.03	0.2	0.22	0.116	0.302
BAK1	0.06	0.02	0.19	0.17	0.129	0.33
GAPDH	0.24	0.03	0.89	0.88	0.139	0.353
MYC	0.14	0.03	0.49	0.31	0.146	0.364
LCK	-0.11	0	0.47	0.35	0.147	0.364
NF2	0.09	0.02	0.33	0.37	0.169	0.411
RICTOR	-0.17	-0.01	0.75	0.7	0.178	0.429
ERBB2	-0.15	-0.01	0.65	0.67	0.181	0.429
EIF4G1	0.04	-0.03	0.73	0.52	0.187	0.435
PRKAA1_PT172	-0.09	0.03	0.56	0.58	0.187	0.435
ARID1A	0.05	0	0.27	0.2	0.201	0.461
PECAM1	-0.03	0.04	0.36	0.34	0.212	0.482
RPTOR	0.07	0.01	0.33	0.35	0.218	0.488
CTNNA1	-0.01	0	0.1	0.13	0.221	0.49
ANXA1	0.28	0.09	0.98	0.69	0.231	0.495
RAB11B	-0.02	0.03	0.27	0.23	0.231	0.495
RAB11A	-0.02	0.03	0.27	0.23	0.231	0.495
AKT3	-0.24	-0.12	0.63	0.59	0.241	0.495
AKT2	-0.24	-0.12	0.63	0.59	0.241	0.495
AKT1	-0.24	-0.12	0.63	0.59	0.241	0.495
TP53BP1	0.08	-0.04	0.65	0.44	0.242	0.495
GAB2	0.16	0.05	0.61	0.5	0.244	0.495
CHEK2	0.12	0.03	0.49	0.41	0.251	0.499
CTLA4	-0.39	-0.17	1.41	1	0.253	0.499
SMAD4	0.05	0.02	0.18	0.17	0.253	0.499
YWHAB	-0.01	0.04	0.28	0.21	0.266	0.506

STAT5A	-0.11	0.01	0.71	0.66	0.268	0.506
GATA3	0.05	0	0.3	0.2	0.269	0.506
CDH2	0.09	0.03	0.36	0.25	0.271	0.506
FASN	0.17	0.04	0.7	0.74	0.272	0.506
BAX	0.13	0.07	0.33	0.36	0.277	0.506
MAPK9	-0.1	-0.04	0.35	0.24	0.278	0.506
ERRFI1	0.04	-0.02	0.37	0.32	0.278	0.506
MTOR	-0.05	0.02	0.46	0.35	0.304	0.549
MS4A1	0.1	0.05	0.28	0.25	0.311	0.557
PREX1	-0.02	0.03	0.32	0.26	0.317	0.559
EEF2	0.02	-0.04	0.33	0.3	0.318	0.559
PARK7	-0.05	0.01	0.39	0.31	0.326	0.562
AKT3_PS473	-0.1	0.02	0.73	0.59	0.332	0.562
AKT2_PS473	-0.1	0.02	0.73	0.59	0.332	0.562
AKT1_PS473	-0.1	0.02	0.73	0.59	0.332	0.562
RPS6KB1	0.04	-0.03	0.49	0.32	0.341	0.573
BID	0.05	0.01	0.26	0.2	0.359	0.598
XRCC5	0.04	-0.03	0.5	0.38	0.366	0.603
MACC1	0.06	0	0.48	0.58	0.369	0.603
SQSTM1	0.23	0.11	0.85	0.74	0.371	0.603
PXN	-0.18	-0.1	0.55	0.55	0.375	0.603
EGFR	0.15	0.06	0.7	0.36	0.38	0.603
XBP1	-0.04	0	0.28	0.27	0.383	0.603
BRCA2	-0.03	0	0.3	0.2	0.386	0.603
NOTCH1	0.02	-0.01	0.26	0.21	0.387	0.603
PDCD1	-0.06	0.01	0.61	0.54	0.39	0.603
CDH3	0	-0.06	0.41	0.33	0.396	0.608
ITGA2	0.12	0.04	0.63	0.42	0.401	0.611
KEAP1	0.09	0.04	0.41	0.51	0.409	0.619
COPS5	0.01	0.02	0.13	0.23	0.413	0.619
AKT3_PT308	-0.04	0.02	0.41	0.35	0.422	0.619
AKT2_PT308	-0.04	0.02	0.41	0.35	0.422	0.619
AKT1_PT308	-0.04	0.02	0.41	0.35	0.422	0.619
MRE11A	0.07	0.03	0.28	0.19	0.432	0.629
SRC	0.07	0.03	0.3	0.32	0.445	0.639
BRAF	-0.12	-0.04	0.65	0.58	0.449	0.639

AXL	-0.04	-0.01	0.27	0.24	0.45	0.639
YWHAE	0.01	0.03	0.17	0.17	0.452	0.639
DIRAS3	0.01	0.02	0.14	0.16	0.468	0.657
ERBB3_PY1289	0.06	0.03	0.21	0.16	0.475	0.662
ACACA	0.11	0.03	0.78	0.62	0.482	0.664
PARP1	0.02	0.05	0.31	0.39	0.483	0.664
E2F1	0.02	0	0.17	0.21	0.489	0.666
HSPA1A	0.24	0.14	0.86	0.83	0.495	0.671
YWHAZ	0.13	0.06	0.63	0.61	0.502	0.675
PRDX1	0.08	0.02	0.59	0.4	0.522	0.687
VHL	-0.24	-0.09	1.45	1.44	0.523	0.687
ETS1	0	0.03	0.28	0.22	0.524	0.687
PIK3R2	-0.09	-0.06	0.32	0.34	0.528	0.687
PIK3R1	-0.09	-0.06	0.32	0.34	0.528	0.687
RAF1_PS338	0.05	0.03	0.21	0.17	0.532	0.687
INPP4B	0.15	0.08	0.79	0.7	0.537	0.688
RAD50	-0.03	-0.05	0.24	0.24	0.546	0.695
MAPK1	-0.15	-0.11	0.46	0.54	0.568	0.72
WWTR1	0.05	0.02	0.35	0.24	0.578	0.728
YAP1	0.05	0.09	0.44	0.36	0.587	0.729
LCN2	0.27	0.14	1.71	1.3	0.592	0.729
TSC1	-0.18	-0.14	0.55	0.58	0.593	0.729
NRAS	-0.03	-0.01	0.18	0.14	0.599	0.729
EEF2K	-0.06	-0.02	0.5	0.44	0.6	0.729
CCND1	0.05	0.03	0.27	0.26	0.608	0.729
G6PD	0.22	0.15	0.9	0.74	0.608	0.729
IGFBP2	0.46	0.34	1.49	1.1	0.61	0.729
FOXO3	0	0.02	0.19	0.18	0.621	0.739
COL6A1	0.12	0.15	0.53	0.53	0.653	0.772
EIF4EBP1_PT37	0.09	0.03	0.78	0.58	0.66	0.775
DVL3	-0.01	-0.03	0.24	0.26	0.672	0.782
TIGAR	0.09	0.06	0.41	0.35	0.674	0.782
BIRC2	0.02	0.01	0.17	0.19	0.679	0.784
ATM	-0.08	-0.12	0.75	0.74	0.733	0.841
ANXA7	0.01	0	0.21	0.19	0.752	0.852
PIK3CA	-0.01	0	0.24	0.18	0.756	0.852

RPS6	-0.01	-0.03	0.5	0.43	0.76	0.852
SYP	0.13	0.18	1.13	0.99	0.76	0.852
SYK	0.02	0.05	0.67	0.48	0.776	0.864
STK11	0.02	0.01	0.25	0.19	0.789	0.874
BCL2L1	0.03	0.04	0.29	0.23	0.798	0.879
BCL2	0.08	0.05	0.74	0.43	0.804	0.88
RET_PY905	0.01	0.02	0.43	0.33	0.813	0.885
PEA15	0.03	0.05	0.4	0.34	0.856	0.925
TP53	0	0.02	0.63	0.53	0.864	0.925
CASP9	0.02	0.01	0.27	0.2	0.868	0.925
TSC2	-0.13	-0.11	0.51	0.49	0.869	0.925
ESR1	0.12	0.11	0.77	0.44	0.872	0.925
NRG1	0.05	0.04	0.28	0.22	0.893	0.94
ACACB_PS79	0.04	0.06	0.68	0.55	0.904	0.94
ACACA_PS79	0.04	0.06	0.68	0.55	0.904	0.94
MAP2K1	0.03	0.02	0.38	0.33	0.906	0.94
EIF4E	0	-0.01	0.2	0.2	0.923	0.952
TUBA1B	-0.07	-0.06	0.94	0.95	0.936	0.957
KDR	-0.01	-0.02	0.72	0.56	0.937	0.957
YBX1	0.05	0.05	0.41	0.6	0.987	0.998
SRSF1	0.03	0.03	0.15	0.18	0.988	0.998
AR	0.03	0.03	0.3	0.25	0.996	0.998
IRS1	0.01	0.01	0.27	0.2	0.998	0.998
